# Predicting Waist Circumference From a Single Computed Tomography Image Using a Mobile App (Measure It): Development and Evaluation Study

**DOI:** 10.2196/38852

**Published:** 2023-12-13

**Authors:** Abderrahmen Masmoudi, Amine Zouari, Ahmed Bouzid, Kais Fourati, Soulaimen Baklouti, Mohamed Ben Amar, Salah Boujelben

**Affiliations:** 1 Surgery Department, Habib Bourguiba University Hospital, Sfax, Tunisia

**Keywords:** waist circumference, computed tomography, abdominal CT, mobile health, health apps, CT, CT scan, CT image, mobile app, app, application, waist, body, body mass, BMI, morbidity, mortality, clinical, tool, prototype, design, obesity, abdominal, usability, validity, medical

## Abstract

**Background:**

Despite the existing evidence that waist circumference (WC) provides independent and additive information to BMI when predicting morbidity and mortality, this measurement is not routinely obtained in clinical practice. Using computed tomography (CT) scan images, mobile health (mHealth) has the potential to make this abdominal obesity parameter easily available even in retrospective studies.

**Objective:**

This study aimed to develop a mobile app as a tool for facilitating the measurement of WC based on a cross-sectional CT image.

**Methods:**

The development process included three stages: determination of the principles of WC measurement from CT images, app prototype design, and validation. We performed a preliminary validity study in which we compared WC measurements obtained both by the conventional method using a tape measurement in a standing position and by the mobile app using the last abdominal CT slice not showing the iliac bone. Pearson correlation, student *t* tests, and Q-Q and Bland-Altman plots were used for statistical analysis. Moreover, to perform a diagnostic test evaluation, we also analyzed the accuracy of the app in detecting abdominal obesity.

**Results:**

We developed a prototype of the app Measure It, which is capable of estimating WC from a single cross-sectional CT image. We used an estimation based on an ellipse formula adjusted to the gender of the patient. The validity study included 20 patients (10 men and 10 women). There was a good correlation between both measurements (Pearson *R*=0.906). The student *t* test showed no significant differences between the two measurements (*P*=.98). Both the Q-Q dispersion plot and Bland-Altman analysis graphs showed good overlap with some dispersion of extreme values. The diagnostic test evaluation showed an accuracy of 83% when using the mobile app to detect abdominal obesity.

**Conclusions:**

This app is a simple and accessible mHealth tool to routinely measure WC as a valuable obesity indicator in clinical and research practice. A usability and validity evaluation among medical teams will be the next step before its use in clinical trials and multicentric studies.

## Introduction

Obesity is a major public health problem worldwide, and the reliance on BMI measurements alone has proven insufficient to help assess obesity-related health risks in patients [1]. Waist circumference (WC) is a simple method to evaluate abdominal adiposity that is easy to standardize. It is also an independent cardiovascular risk factor, with a higher predicting value than BMI [2,3]. However, this measurement is not routinely used in clinical practice.

Recently, a computed tomography (CT) scan estimation became a valid measure of standing WC [4,5]. This method is truly valuable in retrospective studies, where it can be difficult to obtain such measurements. Moreover, conventional WC assessment using a measurement tape can be challenging in patients with intellectual or motor disabilities. However, for a radiologist, this method may require time and training. Therefore, despite its widespread availability and limited cost, using CT images to assess WC is not routinely included in clinical and research practice.

Accordingly, the major aim of this study was to develop a mobile app to overcome these barriers and help clinicians routinely assess WC whenever a CT scan is available.

## Methods

### Overview

The development process involved three stages: determination of the principles of WC measurement from CT images, prototype design, and validation of the developed product.

**Figure 1.**
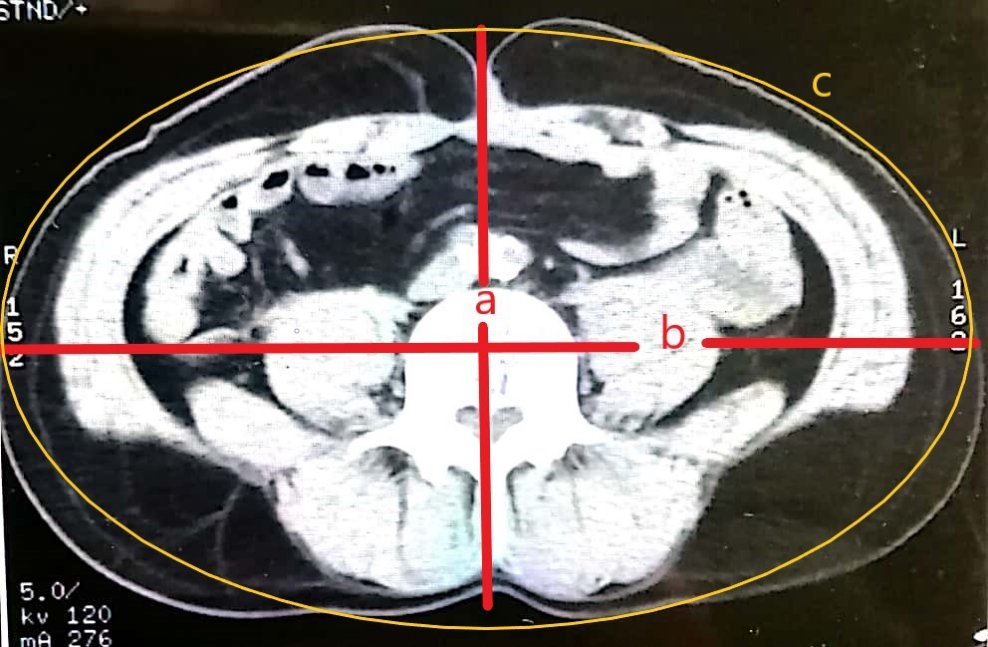
Evaluation of the waist circumference on a computed tomography image using an ellipse perimeter formula. (a) Anterior-posterior diameter, (b) transverse diameter, and (c) ellipse perimeter.

As validated by Ciudin et al [6], the abdominal perimeter was estimated using the formula of the perimeter of an ellipse (Figure 1). In this previous study, there was a good correlation between conventional standing WC measurement and ellipse-estimated WC, with a Pearson test of 0.987 and a mean error of 0.4 cm.

We applied the same formula:

$WC\approx \pi \left\lfloor 3\left(a\;+\;b\right)\;-\;\sqrt{\left(3a\;+\;b\right)\left(a\;+\;3b\right)}\right\rfloor$

“a” being the anterior-posterior diameter and “b” being the transverse diameter. Afterward, we performed WC measurement on 10 healthy candidates using both the conventional tape method and the ellipse formula. We then used a simple linear regression analysis to adjust the final WC formula to the gender of the patient.

After confirming the app requirements (ellipse formula, required measurements, final formula applied to gender and the needed parameters, and organization of the steps required by the physician to ameliorate the user experience), we initiated the design and development of the app. After preparing the app prototype, we performed a preliminary validity study including 20 patients selected retrospectively based on the existence of a previous WC measurement and CT scan images in their file. We compared the conventional WC measurement (cWC) method to the mobile app–based WC measurement (mWC) method based on CT scan images.

The first measurement was done using a measuring tape placed horizontally around the patient’s abdomen just above the iliac crest as recommended by the National Institutes of Health National Institutes of Health National Institutes of Health guidelines [7]. It was done in a standing position, at the end of a normal expiration. The second measurement was performed with the mobile app. Using the camera of the phone, the app employused the last slice of the CT scan image, on the last slice, from cranial to caudal, not showing the iliac bone.

Measurements were expressed as mean ± (SD)standard deviation and range. Data were collected and analyszed using SPSS 20 software (SPSS Inc., Chicago, IL, USAIBM Corp). Abdominal obesity was defined by waist circumferenceWC measurements of >102 cm (~40 inches) and >88 cm (~35 inches) for men and women, respectively [8]. Student t test, Pearson correlation, Q-Q plot, and the Bland-Altman analysis were used. P values <inferior to 0.05 were considered statistically significant from a statistical point of view. In order tTo perform a diagnostic test evaluation, we also analyszed the mWC accuracy in detecting abdominal obesity.

At the end of the design stage, the app was demonstrated in several team meetings, which led to further modifications.

### Patient and Public Involvement

The patients were not involved in setting the research question or the outcome measures, designing or implementing the study, or reporting or disseminating the research. Additionally, the public did not participate in the design, implementation, reporting, or dissemination plans of this study.

### Ethical Considerations

Personal data have been respected. This study was approved by the ethics committee at Habib Bourguiba University Hospital in Sfax (Ref CE-03-2022).

## Results

**Figure 2.**
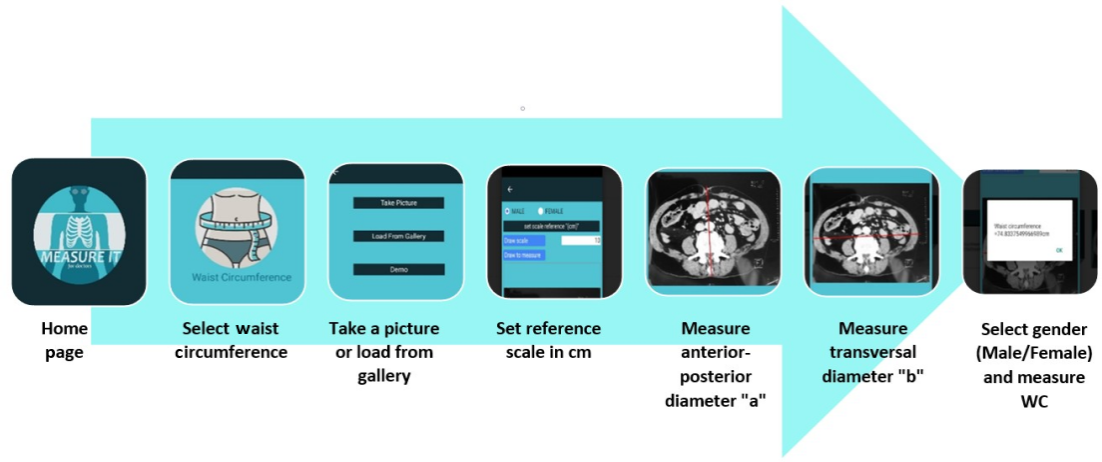
Illustration of the flow of the app. WC: waist circumference.

Following the design principles and requirements, the prototype of the app was developed and named Measure It. The flow of the app was designed to be simple and productive to ensure quality interaction between the app and the visitor (Figure 2). A demo video was provided in the app to facilitate its use.

The preliminary validation study included 20 patients. It included 10 men and 10 women. The mean age was 54 (SD 17) years. The mean BMI was 26 (SD 4; women: mean 27.8, SD 2.7; men: mean 24.2, SD 4.4). The mean cWC was 93.7 (SD 12.6, range 68-122; women: mean 95.1, SD 11.9; men: mean 92.3, SD 13.8) cm. The most common reason why patients (n=10) underwent an abdominal CT scan was for biliary stone disease.

**Figure 3.**
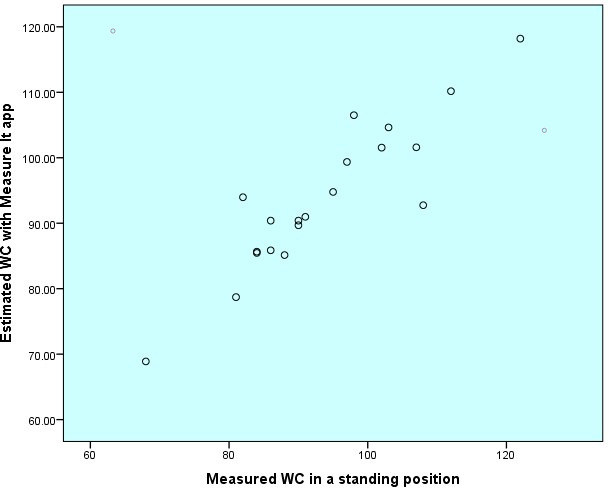
Q-Q plot of estimated WC versus measured WC. WC: waist circumference.

The comparison between cWC and mWC showed a good correlation (Pearson *R*=0.90). The student *t* test showed no significant difference between the two measurements (*P*=.98). We also compared the two measurements using a Q-Q dispersion and a Bland-Altman plot. The analysis graphs can be found in Figures3  4. The Q-Q plot showed good overlap with some dispersion of extreme values. The Bland-Altman analysis showed a mean difference of 0.03 cm (95% CI –2.46 to 2.53) between the two measurements ().

**Figure 4.**
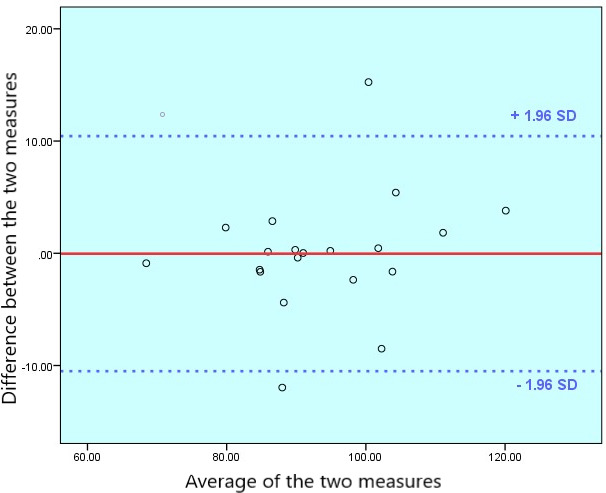
Bland-Altman plot of the differences between the standing and app-estimated waist circumferences.

We have also performed a diagnostic test evaluation regarding the accuracy of the mWC in detecting abdominal obesity. Results were adjusted to the prevalence of abdominal obesity (59%) [9]. The analysis showed an accuracy of 83% when using mWC to detect abdominal obesity (Table 1).

**Table 1.** Diagnostic test evaluation for mobile app waist circumference.

Statistic	Value (95% CI)	
Sensitivity (%)	72.73 (39.03-93.98)	
Specificity (%)	100.00 (66.37-100.00)	
Positive predictive value^a^ (%)	100.00	
Negative predictive value^a^ (%)	71.82 (49.26-86.99)	
Accuracy^a^ (%)	83.91 (60.82-96.28)	

a These values are dependent on abdominal obesity prevalence [9].

## Discussion

### Principal Findings

Guidelines for the management of obesity from several professional societies recognize the importance of measuring WC in the context of risk stratification for future cardiometabolic morbidity and mortality [3,10-13]. Moreover, WC is gaining significant importance among surgeons since abdominal obesity has a growing value in preoperative risk assessment for morbidity and mortality in different surgeries [14-19].

We developed a prototype of the mobile app Measure It to accurately estimate WC using CT scan images. The app was developed based on a validated method [5,6] measuring WC using CT scan cross-sectional images. To our knowledge, this is the first mobile app that helps physicians estimate WC. The app was designed to be a simple and accessible tool with the purpose of routinely including this valuable obesity parameter in clinical and research practice. One of the most valuable advantages of our app is its usability in retrospective studies. WC measurements mostly do not exist in patient observations. However, CT scan slides or images are often available. Moreover, the simplicity of the app may reduce the time required for physicians to assess WC [20]. Conventional tape measuring is sometimes not possible, particularly for patients who are disabled. Additionally, for a radiologist, the conventional CT scan method requires training and can be more or less time-consuming. Eventually, being simple, accessible, and reproducible, the app may reduce the technology barriers for nontech physicians since smartphones are commonly available even in low- and middle-income countries [21].

As a screening tool for abdominal obesity, this attribute may be beneficial, especially in retrospective studies. With an accuracy of 83% compared to the conventional method, the mobile app method is reliable. However, the accuracy of WC measurement may be altered in some cases. This may be due to a measurement error in the conventional method or to particular body shapes and extreme values of WC.

One major problem with currently available mobile health apps is that few are established with strong research evidence [22,23]. Measure It is developed based on a strong statistical analysis, even though it needs to be validated in a prospective study.

The main limitation of this study is the small sample size used to validate the app. We consider this validity study as a preliminary validation that needs to be confirmed. Therefore, we would expect our app to be ready for clinical use to a certain degree.

Another limitation is that the small screen on the smartphone makes it difficult to precisely set reference scales and perform measurements on a CT scan image. However, the zooming functionality makes the app’s accuracy very sufficient. To overcome this problem, we plan to develop a second prototype app with artificial intelligence technology to automatically detect the reference scale and make the essential measurements on the image without the user interfering.

### Conclusions

In this study, we developed a prototype of a mobile app for estimating WC for physicians. Being simple, available, and reproducible, this app has the potential to positively affect the quality of data in future research. Usability and validity evaluation among medical teams will be the next step before its use in clinical trials and multicentric studies.

## Abbreviations

CT: computed tomography
cWC: conventional waist circumference measurement
mHealth: mobile health
mWC: mobile app–based waist circumference measurement
WC: waist circumference
